# Accelerated mapping of electronic density of states patterns of metallic nanoparticles via machine-learning

**DOI:** 10.1038/s41598-021-91068-8

**Published:** 2021-06-02

**Authors:** Kihoon Bang, Byung Chul Yeo, Donghun Kim, Sang Soo Han, Hyuck Mo Lee

**Affiliations:** 1 Department of Materials Science and Engineering, Korea Advanced Institute of Science and Technology (KAIST), 291 Daehak-ro, Yuseong-gu, Daejeon, 34141 Republic of Korea; 2 Computational Science Research Center, Korea Institute of Science and Technology (KIST), 5 Hwarang-ro 14-gil, Seongbuk-gu, Seoul, 02792 Republic of Korea

**Keywords:** Condensed-matter physics, Materials for energy and catalysis, Nanoscale materials, Theory and computation, Nanoscale materials

## Abstract

Within first-principles density functional theory (DFT) frameworks, it is challenging to predict the electronic structures of nanoparticles (NPs) accurately but fast. Herein, a machine-learning architecture is proposed to rapidly but reasonably predict electronic density of states (DOS) patterns of metallic NPs via a combination of principal component analysis (PCA) and the crystal graph convolutional neural network (CGCNN). With the PCA, a mathematically high-dimensional DOS image can be converted to a low-dimensional vector. The CGCNN plays a key role in reflecting the effects of local atomic structures on the DOS patterns of NPs with only a few of material features that are easily extracted from a periodic table. The PCA-CGCNN model is applicable for all pure and bimetallic NPs, in which a handful DOS training sets that are easily obtained with the typical DFT method are considered. The PCA-CGCNN model predicts the R^2^ value to be 0.85 or higher for Au pure NPs and 0.77 or higher for Au@Pt core@shell bimetallic NPs, respectively, in which the values are for the test sets. Although the PCA-CGCNN method showed a small loss of accuracy when compared with DFT calculations, the prediction time takes just ~ 160 s irrespective of the NP size in contrast to DFT method, for example, 13,000 times faster than the DFT method for Pt_147_. Our approach not only can be immediately applied to predict electronic structures of actual nanometer scaled NPs to be experimentally synthesized, but also be used to explore correlations between atomic structures and other spectrum image data of the materials (e.g., X-ray diffraction, X-ray photoelectron spectroscopy, and Raman spectroscopy).

## Introduction

Nanoparticles (NPs) are of great scientific interest because they often show unexpected physical and chemical properties resulting from their quantum confinement effect^1,2^ or high surface area^3,4^. This leads to various applications of NPs, such as quantum dots^5–7^, magnetic^8,9^ or bio-^10–13^ materials, and catalysis^3,14–21^. As a key feature to determine the properties of NPs, an electronic structure such as electronic density of states (DOS) has been usually considered, where the electronic structure significantly depends on the sizes and shapes of the NPs although the elements constituting the NPs are identical^9,17,20,22–26^.

First-principles density functional theory (DFT) calculations have been mainly utilized to predict DOS patterns of NP structures. In particular, the plane-wave (PW) basis has been employed for metallic NP systems despite its extremely high computational cost for large finite-size systems. Moreover, NP structures require a much higher computational cost than bulk or slab structures. In the PW–DFT framework, it is necessary that the entire simulation box, including the vacuum space, must be filled with PWs, seriously reducing the computational speed^27^. In this regard, the fast but accurate electronic structure calculation for metallic NPs still remains challenging.

To bypass the first-principles framework, a machine-learning (ML) approach has been recently pursued^28–42^. In particular, Chandrasekaran et al.^29^ developed a neural network (NN) model for the prediction of DOS patterns and showed that its computational cost was linearly scaled with system size (*N*) [O(*N*)], while the DFT method was scaled as O(*N*^2^). With a similar aim, Yeo et al.^30^ developed an ML scheme based on principal component analysis (PCA) and successfully applied it to bulk and slab structures of multicomponent metallic systems. Moreover, it showed a computational cost independent of the system size. Despite such success, the scheme predicts the DOS pattern of a test system via a linear interpolation between the two training systems that is most similar to the test composition, which likely reduces the versatility of the scheme. When mapping the DOS patterns of materials, it is important to appropriately reflect the local environments of each atom in the structures because the DOS patterns are sensitive to the local atomic environment.

Metallic NP structures can be regarded as consisting of core and shell regions. Here, although the core region can be treated as a bulk structure, the shell region is an assembly consisting of surface atoms with various coordination numbers, which motivates us to improve the previous PCA-based method by more elaborately learning the local environments of atoms in NPs when predicting their DOS patterns. Xie and Grossman^43^ reported a crystal graph convolutional neural network (CGCNN) framework enabling a universal and interpretable representation of crystalline materials. This model converts atomic structures in bulks to graphs, and then the graph fingerprints learn the local environments of atoms by an additional CNN process. Compared with other ML frameworks widely used in materials science field such as Gaussian Process Regression^37,39,40^ or LSBoost^36,38^, the CGCNN has several advantages when used to predict DOS patterns of NPs. First, the CGCNN can account for the local chemical environment of atoms which can sensitively affect the DOS patterns during the learning process via convolution of the constructed graphs. Also, there is no limitation regarding atomic structure (number of atoms, number of elements, shape, etc.) for the input of the CGCNN framework, thus, NP structures with various size and shape and corresponding DOS patterns can be used as datasets. Moreover, the CGCNN provides reasonable accuracy even with just periodic-table level properties as features, indicating that no additional quantum calculation is needed in predicting the DOS pattern. Recently, we also demonstrated that the CGCNN framework can be extended to slab structures^21^. These facts reveal that the CGCNN is readily applicable for representing atomic structures of NPs, and a combination of PCA and the CGCNN is expected to provide a reasonable and fast mapping of DOS patterns of NPs.

In this work, we propose an ML paradigm to predict DOS patterns (both of shapes and of values) of metallic NPs through a combination of PCA and the CGCNN, where the model is learned with DOS patterns of small-sized NPs (e.g., Au_19_) that are not time-consuming to obtain with the state-of-the-art DFT calculations. Within the PCA-CGCNN framework, one can predict DOS patterns for not only pristine NPs but also alloyed ones with a small loss of accuracy compared to DFT calculations, where effects of the sizes and shapes of metallic NPs have also been explored. Moreover, the method shows a computational cost nearly independent of the system size.

## Computational details

### NP structures for the DOS database

**Figure 1:**
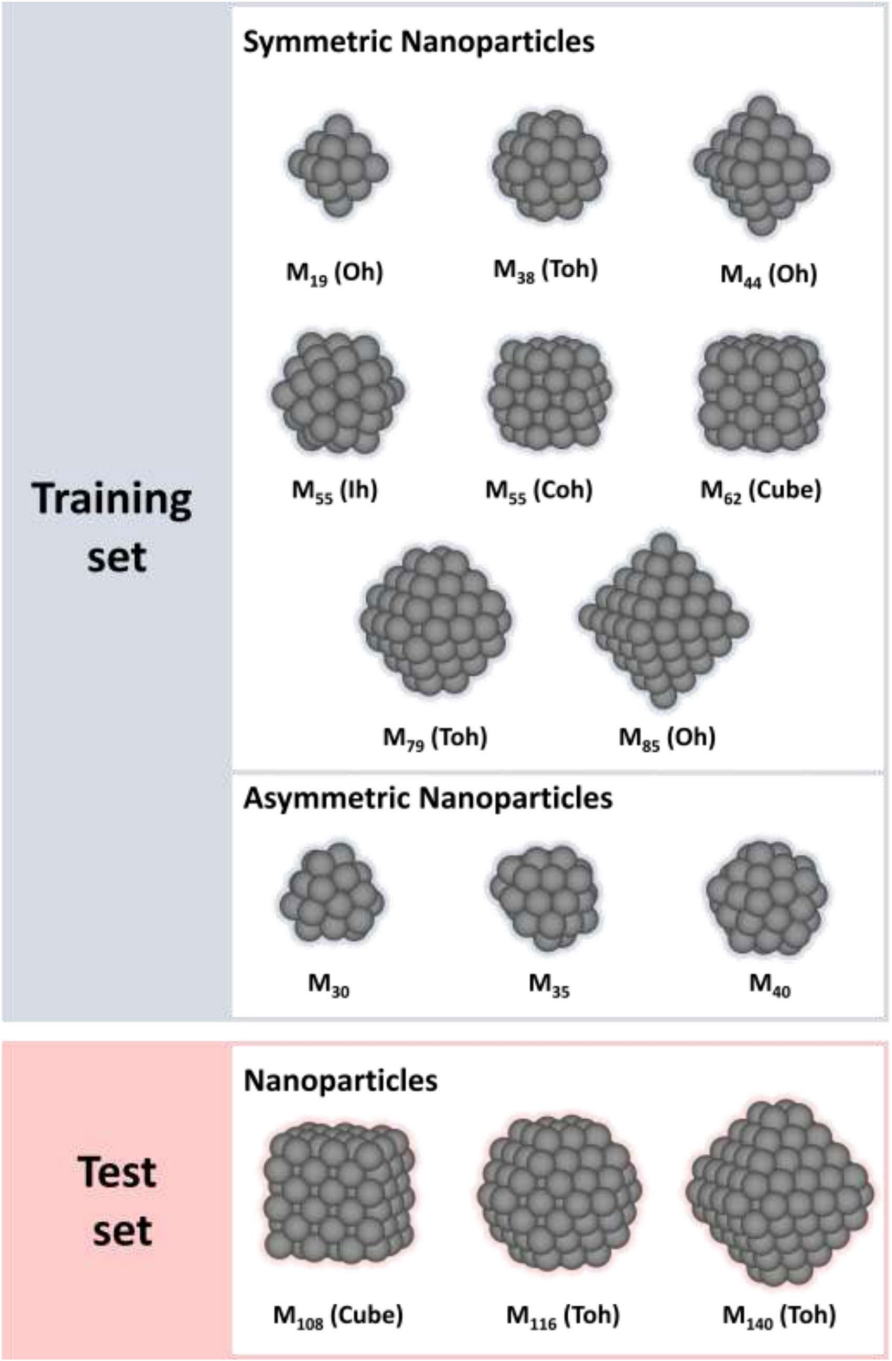
Training and test datasets for DOS prediction of NPs. In the NP structures, COh, Ih, Oh, TOh, and Cube indicate cuboctahedral, icosahedral, octahedral, tetraoctahedral, and cubic structures, respectively.

Figure 1 shows various NP structures used in the training and test sets. We prepared NPs composed of 19–140 atoms with symmetric shape. These NP structures have been studied in various catalyst researches^22,44,45^ as they represent specific surface properties and size effects. The NPs with symmetric shapes were constructed by Atomic Simulation Environment module^46^ and DFT ionic relaxation. In addition, to consider local environment effects such as strains and defects, NPs with asymmetric shapes (40, 45, and 50 atoms) were also considered, where the asymmetric NPs were constructed by molecular dynamics simulations using the Large-scale Atomic/Molecular Massively Parallel Simulator (LAMMPS) package^47^ and embedded atom method potentials^48^. The detail is described in Supplementary material.

### DFT calculations

To obtain electronic DOS patterns of the training and test NP structures, spin-polarized DFT calculations with plane-wave basis sets were carried out using the Vienna Ab initio Simulation (VASP) package^49,50^. We used the generalized gradient approximation with the revised Perdew-Burke-Ernzerhof functional^51,52^ to describe the exchange–correlation energy of electrons. Ionic cores were treated by the projector-augmented wave (PAW) method^53^. The plane-wave cutoff was set to 520 eV, and the convergence criteria for electronic structure and atomic geometry were 1.0 × 10^–4^ eV and 0.03 eV/Å, respectively. The Brillouin zone was sampled using a Monkhorst–Pack *k*-point mesh^54^, and the *k*-point sampling was set to 1 × 1 × 1 for NP structures. A large vacuum spacing > 20 Å was used for NP structures to prevent interslab interactions. The DOS patterns were normalized by the number of atoms in the system and were shifted to set the Fermi level (E_f_) to 0 in the pattern.

### Details of PCA

Because the mathematical dimension of a DOS pattern is very high (e.g., 3000 energy levels × DOS values of 4-byte floats in our DFT calculations), it is very challenging to map the DOS pattern with only common material features as an input information, such as the number of atoms, composition, and lattice parameter. Thus, it is necessary to reduce the DOS patterns to a low-dimensional vector. To do this, we applied PCA in this work. Prior to the analysis, the training DOS data were regularized into 200-dimension vectors in the energy range of − 8 to 3 eV relative to the Fermi level (0 eV) by interpolation, where the energy range was divided into 200 energy windows. The 200-dimensional DOS vectors were represented with the DOS values themselves at each energy window, although Yeo et al.^30^ converted a DOS pattern to a digital image vector with M × N entries (black and white pixels), implying that the DOS image vector can include information irrelevant to the original DOS values. We standardized the DOS vectors of the training data by obtaining the normalized matrix $\mathbf{Y}$, in which the *i*th energy window (${\mathbf{y}}_{{\varvec{i}}}$) of $\mathbf{Y}$ is ${\mathbf{x}}_{{\varvec{i}}}-\overline{\mathbf{x}}$, where $\overline{\mathbf{x}}$ is the mean of each column vector of $\mathbf{X}$. Then, we calculated the principal components (PCs) or eigenvectors, ${\mathbf{u}}_{p}$**=**${({u}_{1},{u}_{2},\dots ,{u}_{200})}_{p}$, and the corresponding eigenvalues, ${\uplambda }_{p}$, were calculated by the covariance matrix, **S** = **Y**^**T**^**Y**, and Eq. (1).

1 $$\mathbf{S}{\mathbf{u}}_{{\varvec{p}}}={\lambda }_{p}{\mathbf{u}}_{{\varvec{p}}}$$

The original DOS image vector **x** can be reconstructed as follows:

2 $$\mathbf{x}\cong \sum_{p=1}^{P}\left({\mathbf{y}}^{{\varvec{T}}}{\mathbf{u}}_{{\varvec{p}}}\right){\mathbf{u}}_{{\varvec{p}}}+\sum_{p=1}^{P}\left({\overline{\mathbf{x}}}^{{\varvec{T}}}{\mathbf{u}}_{{\varvec{p}}}\right){\mathbf{u}}_{{\varvec{p}}}=\sum_{p=1}^{P}{\mathrm{\alpha }}_{\mathrm{p}}{\mathbf{u}}_{{\varvec{p}}}$$

where P is the number of used PCs and *p* is their index. Thus, coefficient ${\alpha }_{p}$ of the eigenvectors can be computed by ${\mathbf{y}}^{\mathrm{T}}{\mathbf{u}}_{p}+{\overline{\mathbf{x}}}^{\mathrm{T}}{\mathbf{u}}_{p}$, corresponding to the coordinate values on the linear subspace that is composed of PCs. In other words, the signal vector **α** = (α_1_, α_2_, α_3_,…, α_P_)^**T**^ can be defined as a one-to-one correspondence vector of **x**. Similar to Yeo et al.^30^, we implemented our own Python code to perform PCA as we described above. NumPy package was used for matrices operation during the PCA process. However, in our new scheme, we extracted the signal vectors for partial DOS patterns of each atom in NP structures by the PCA process, while Yeo et al.^30^ considered total DOS patterns.

### DOS pattern similarity

The DOS pattern similarity of our PCA-CGCNN model was calculated through two values. One is the coefficient of determination (*R*^*2*^) of the DOS pattern, which is defined as follows:

3 $${R}^{2}=\frac{{\sum }_{m}{\left(\rho \left({E}_{m}\right)-{\rho }^{^{\prime}}\left({E}_{m}\right)\right)}^{2}}{{\sum }_{m}{\left(\rho \left({E}_{m}\right)-\overline{\rho }\right)}^{2}}$$

And, the other is mean absolute error (MAE), which is defined as follows:

4 $$\mathrm{MAE}=\frac{{\sum }_{m}|\rho \left({E}_{m}\right)-{\rho }^{^{\prime}}\left({E}_{m}\right)|}{m}$$

where ρ and ρ′ are the DOS patterns calculated by DFT and predicted by our PCA-CGCNN model, respectively, and $\overline{\uprho }$ is the average of DOS patterns calculated by DFT, and m is the number of energy windows.

## Results and discussion

### Architecture of the PCA-CGCNN model

**Figure 2:**
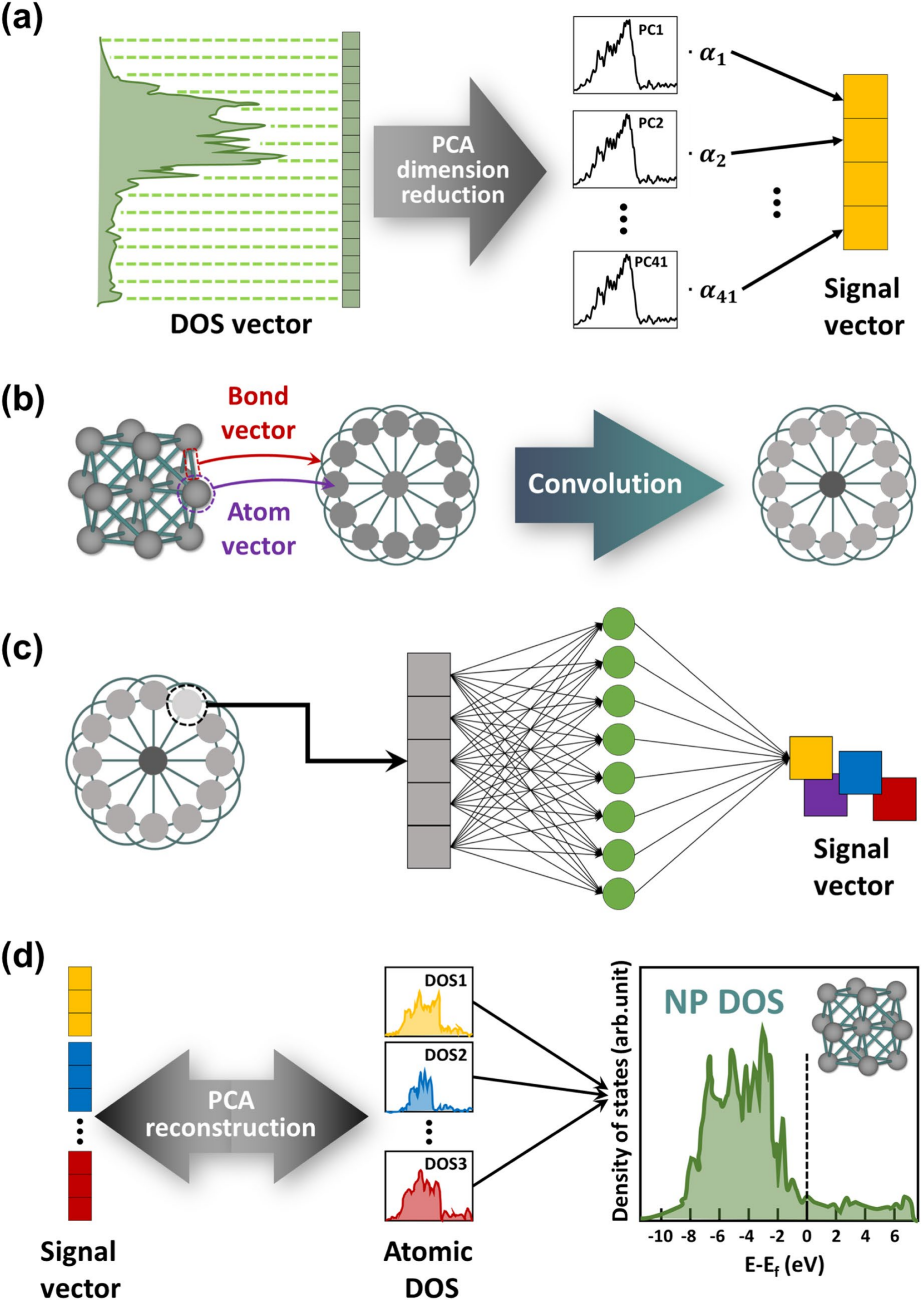
Illustration of the PCA-CGCNN architecture. (**a**) Dimension reduction of DOS vector by principal component analysis (PCA). The coefficients (α) became signal vector. (**b**) Construction of the crystal graph (CG) of NP structures and the structure of the convolutional neural network (CNN) on top of the CG. NP structures are converted to graphs with nodes and edges representing atoms and bonds, respectively. Then, the CNN processes are followed to reflect the local environments of each node in the CG. (**c**) ) Determination of signal vectors. After the CGCNN process, the new graph vector for each atom is fully connected with a signal vector for the DOS representation of each atom by neural networks. (**d**) DOS representation. With the signal vector obtained from the CGCNN, atomic DOS patterns are reconstructed on the basis of PCA. The sum of each atomic DOS pattern produces a total DOS pattern of the NP.

In predicting the DOS pattern of a test system, we used the CGCNN^43^ model to determine the new signal vector for the test system (Fig. 2). Following the original CGCNN scheme, graphs for NP structures were constructed with nodes and edges, in which the nodes and edges represented atoms and bonds, respectively. In the graph, the atom vector ${{\varvec{v}}}_{{\varvec{i}}}$ was encoded in a one-hot manner only with features that were readily available from the periodic table of elements (e.g., period/group number, melting temperature, etc.) due to their categorical property. The bond vector ${{\varvec{u}}}_{({\varvec{i}},{\varvec{j}})}$ was also encoded in a one-hot manner based on the bond length between atoms, in which the bond between the ith and jth atoms was defined only if d_i,j_ < r_i_ + r_j_ + Δ, where d_ij_ is a distance between the atoms i and j, and r_i¸_and r_j_ are the radii of atoms i and j, respectively, with the tolerance Δ = 0.25 Å. A list of the input features for the atom and bond vectors and their ranges/categories is available in Supplementary Tables S1 and S2.

Then, CNN processes were performed on top of the constructed graph, which consisted of a sequence of convolutions. The convolution functions first concatenated neighbor vectors ${z}_{\left(i,j\right)}^{t}={\nu }_{i}^{t}\oplus {\nu }_{j}^{t}\oplus {u}_{\left(i,j\right)}$ and then performed convolutions to update each atom vector, as follows:

5 $${\upnu }_{i}^{\mathrm{t}+1}={\nu }_{i}^{t}+\sum_{j}\sigma ({z}_{\left(i,j\right)}^{t}{W}_{f}^{t}+{b}_{f}^{t})\odot g({z}_{\left(i,j\right)}^{t}{W}_{s}^{t}+{b}_{s}^{t})$$

where *t* denotes the number of convolutional layers; ⊕ denotes concatenation; $\odot$ denotes element-wise multiplication; *σ* is a sigmoid function; g is the rectified linear unit (ReLU) function; and ${W}_{f}^{t}$, ${W}_{s}^{t}$ and ${b}_{f}^{t}$, ${b}_{s}^{t}$ are convolutional weight metrics and biases of the *i*th layer, respectively. After the convolution, the atom vectors for each atom learned with surrounding atoms and bonds in the NP structures can be extracted. Then, the learned atomic vectors were fully connected with the atomic signal vectors obtained from PCA via a neural network, in which the processes were performed for each atom in the training NP systems. Then, the total DOS pattern for a given NP structure was reconstructed through a summation of the partial DOS patterns mapped by the PCA-CGCNN architecture. The proposed architecture was implemented in the Python code with the TensorFlow framework (version 1.13.1) and NumPy package.

### Hyperparameter optimization of the PCA-CGCNN model

The hyperparameters of the PCA-CGCNN model were thoroughly tested. One of the most important hyperparameter is the size of the output node in the CGCNN model, which is identical to the number of used PCs in PCA. As the number of used PCs increases during the PCA process, the loss of information decreases. However, the number of parameters in CGCNN increases as the size of the output node increases, thus it would be challenging to train the CGCNN model with a unsufficient number of data. To find a suitable number of the output node, we calculated the ratio of information (reconstruction rate) and the MAE for the DOS signal vectors of NPs as a function of the PC number, where Au NPs were considered as an example. As shown in Supplementary Fig. S2, the lowest MAE was observed when 41 PCs were used and the ratio of information at the point showed a reasonable value of 0.969.

The other hyperparameters were optimized in a similar manner. The optimized values shown in parentheses are as follows: the number of convolution filters and layers (1 filter, 2 layers), initial learning rate (1 × 10^–3^), exponentially decaying learning rate (0.97 for every 100 epochs), nodes of the hidden layers (3 layers with 63 nodes/layer), standard deviation of normally distributed random initial weights (0.01), batch size (32), and total number of epochs (1000). The loss function was set as the mean square error (MSE).

As the number of training data is quite small, an overfitting problem would be likely issued during training convolutional neural network. Similar to Xie et al.^43^ and Kim et al.^21^, the dropout^55^ and L^2^ regularization were applied to overcome the overfitting, where the dropout rate and L^2^ regularization coefficients were 0.1 and 10^–5^, respectively. As shown in Fig. S10, the MAE difference between the training and validation set become much lower by considering the dropout and regularization. Therefore, we can conclude that our model readily overcomes the overfitting via the dropout and regularization.

For atom vectors, we considered the following features; group number, period number, radius, electronegativity, ionization energy, electron affinity, volume, atomic weight, melting temperature, boiling temperature, density, Z_eff_, polarizability, resistivity, heat capacity, the number of valence electrons, and the number of *d*-electrons. A list of the input features for the atom and their ranges/categories is available in Supplementary Table S1. To select appropriate features for the atom vector, we calculated the MAE for the signal vectors of Au NPs as a function of the number of features (Supplementary Fig. S2). For the cost efficiency, the best feature set was fixed by increasing the number of features. For example, the lowest MAE for the use of one feature was found with an atomic weight; thus, atomic weight was always included in the feature sets of the subsequent tests. From this optimization, the lowest MAE was found when only one feature (atomic weight) were used. Therefore, in this work, we used the one feature for representing the atom vectors of Pt, Au, and Pd in the CGCNN. For bond vectors, we categorized distances between two atoms in the range of 2.4 to 3.4 Å into 40 dimensions (Supplementary Table S2).

### DOS prediction with PCA-CGCNN model: pure NPs

**Figure 3:**
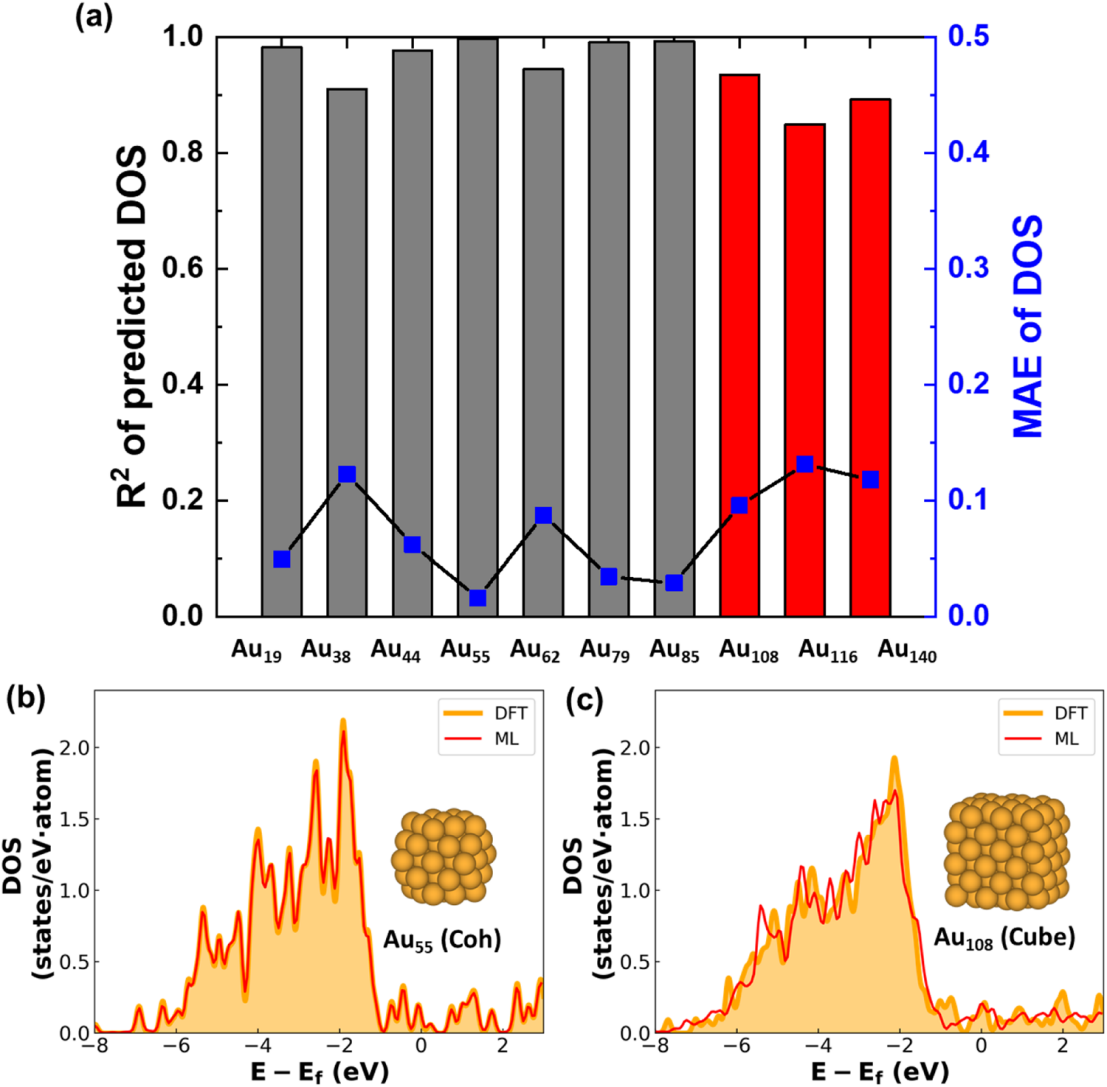
PCA-CGCNN performance on Au NPs. (**a**) The DOS pattern similarity (R^2^ and MAE) of our PCA-CGCNN model compared to DFT methods. Here, pure Au NPs are considered. Bars indicate R^2^ value and blue squares indicate MAE. Gray bars indicate training data, and red bars indicate test data. (**b**,**c**) Comparison of DOS patterns for Au_85_ (**b**) and Au_108_ (**c**) NPs predicted by the DFT method (orange) and the PCA-CGCNN model (red).

To validate our PCA-CGCNN model, we started with pure metallic NPs (Au, Pd, and Pt). A comparison of the DOS patterns of Au NPs obtained from the DFT method and the PCA-CGCNN model is shown in Fig. 3. For the Au NPs, the similarities (R^2^ and MAE) of the DOS patterns reconstructed from the PCA-CGCNN model are in the ranges of 0.911 ~ 0.998 (R^2^) and 0.050 ~ 0.123 (MAE) for the training systems and 0.850 ~ 0.936 (R^2^) and 0.096 ~ 0.137 (MAE) for the test systems (Fig. 3a). The similarity is overall increased as the NP size becomes larger, which can be understood from the fact that a larger NP has lower surface fraction. Because surface atoms in NPs have different chemical environments (e.g., coordination numbers and bond lengths) than core atoms, it is likely more challenging to map DOS patterns of the smaller NPs in a given dataset. Considering that the computation cost of the DFT calculation is significantly increased with the size of NPs^29,56^, the superior prediction capability of the PCA-CGCNN model for larger NPs becomes a strong advantage of this model in terms of computational efficiency. In Fig. 3b,c, the DOS patterns of Au_55_ and Au_108_ NPs are shown, where the DOS similarities (R^2^) of the PCA-CGCNN model are 0.998 and 0.936, respectively. Indeed, our ML scheme reasonably reproduces the DFT pattern; in particular, the peak positions are very well matched, although only a handful of training structures are considered. For pure Pt and Pd NPs, our ML scheme demonstrates similar predictive abilities to those observed in the Au NPs (Supplementary Figs. S3 and S4), which clearly validates our PCA-CGCNN method.

**Figure 4:**
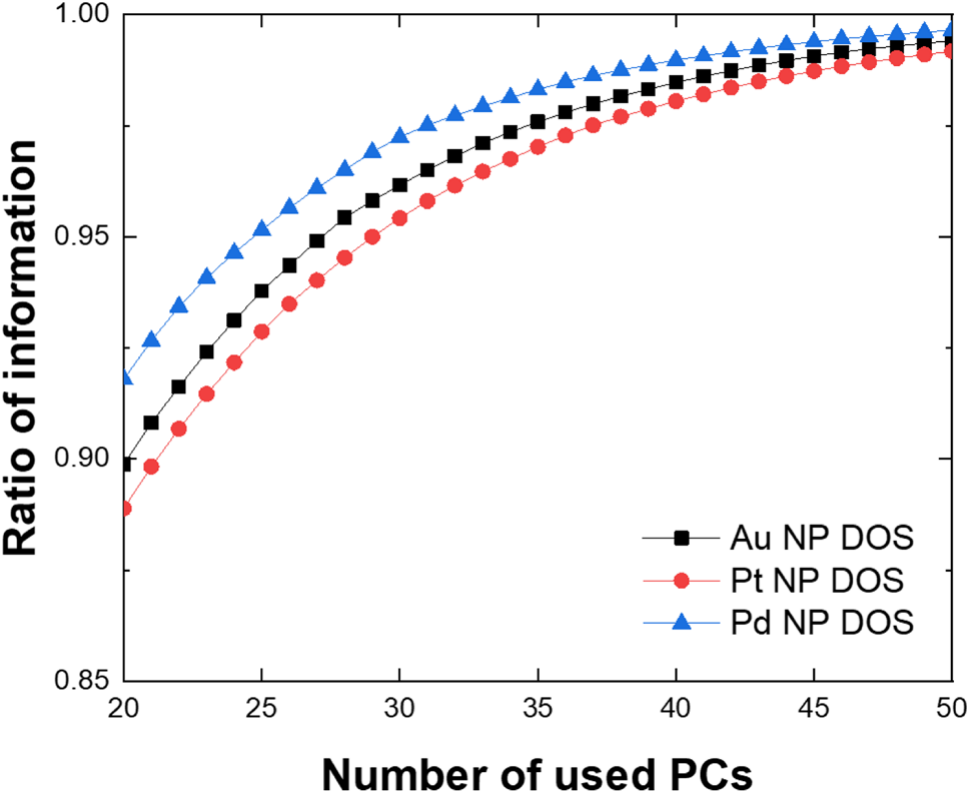
PCA of Au, Pt, and Pd NPs. The ratios of information for DOS patterns of Au, Pt, and Pd NPs DOSs in PCA analysis. Here, the ratio of Pd NP is higher than those of Au and Pt NPs.

Interestingly, the prediction performance for Pd NPs is slightly better than Au and Pt NPs. To unveil the reason, we compared the ratios of information (reconstruction rates) for the DOS patterns of Au, Pt, and Pd NPs during PCA process and found that the reconstruction rate for Pd NPs is higher than Au and Pt NPs (Fig. 4), which indicates that the DOS patterns of Pt and Au NPs are more dispersed than those of Pd NPs. This is more clearly observed by comparing the average of standard deviation for the DOS value at each energy level. Indeed, the value for Pd NPs is 0.238, which is lower than those of Au (0.253) and Pt (0.254) NPs. The difference comes from the polarizability of elements. As the electric dipole polarizability of Pd (26.1 a.u.) is smaller than Au (36 a.u.) and Pt (48 a.u.)^57^, the electrons of Pd are relatively less sensitive to the local environment in the NP structures than those of Au and Pt and thus the DOS patterns of Pd NPs would be less changed in comparison to those of Au and Pt, which matches with the trend observed in PCA (Fig. 4). Accordingly, the PCA-CGCNN method is sensitive to the dispersity of DOS and shows a better performance for Pd NPs than Au and Pt NPs.

### DOS prediction with PCA-CGCNN model: bimetallic core@shell NPs

**Figure 5:**
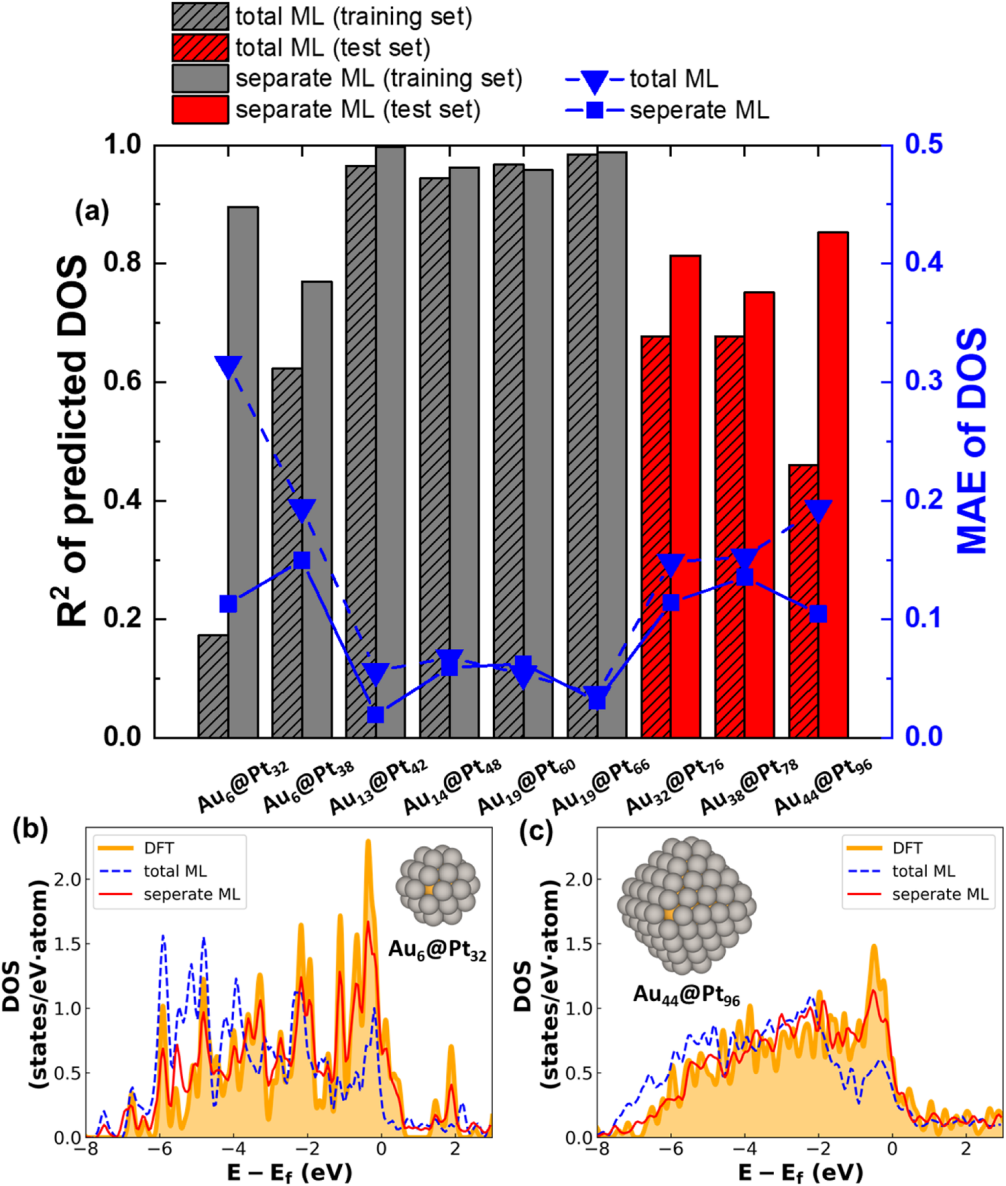
PCA-CGCNN performance on Au@Pt bimetallic NPs. (**a**) The DOS pattern similarity (R^2^ and MAE) of our PCA-CGCNN model compared to DFT methods. Here, bimetallic Au@Pt NPs are considered. Bars indicate R^2^ value and blue squares indicate MAE. (**b**,**c**) Comparison of DOS patterns for Au_6_@Pt_38_ (**b**) and Au_32_@Pt_76_ (**c**) NPs predicted by the DFT method (yellow) and the PCA-CGCNN model (blue = total learning and red = separate learning).

To examine the transferability of our PCA-CGCNN method to bimetallic systems, Pd–Pt and Pt–Au binary core@shell systems. In training DOS patterns for the systems, we used training DBs including pure and alloyed systems (Supplementary Fig. S5). When learning DOS patterns in each bimetallic system, we first applied PCA for atoms in training systems together, hereafter called total ML. The ML model for the Au@Pt systems provides low DOS similarities. Even for Au_6_@Pt_32_ in the training set, the DOS similarity (R^2^) value is so low that it is only 0.173. (Fig. 5). For other core@shell-type systems, similar behaviors are observed (Supplementary Figs. S6–S8).

To improve the prediction ability of our PCA-CGCNN model, we propose a separate learning scheme during the PCA algorithm. For example, when predicting the DOS patterns of Pt–Au NP systems, the original PCA-CGCNN model was simultaneously trained with the DOS patterns of Pt, Au, and bimetallic Pt–Au NPs in the training set, and then the DOS patterns were predicted or reconstructed by the single model. However, in the separate learning scheme, the DOS patterns are individually trained for each atom, i.e., one model is trained with the atomic DOS patterns of Pt atoms in pure Pt and Pt–Au NPs, and another model is trained with those of Au atoms in pure Au and Pt–Au NPs. In the prediction process, the patterns of Pt atoms in bimetallic NPs are mapped with the Pt DOS-trained model, and the patterns of Au atoms are mapped with the Au DOS-trained model. Then, the mapped partial DOS patterns are summed to obtain the total DOS of each NP. In Fig. 5, the prediction ability of the PCA-CGCNN model for the Au@Pt NPs is significantly improved by the separate learning scheme. The DOS similarities (R^2^ values) of Au_6_@Pt_32_, Au_6_@Pt_38_, and Au_44_@Pt_96_ are 0.173, 0.622, and 0.460 from the total ML scheme, respectively; however, the separate ML scheme leads to 0.896, 0.770, and 0.853, respectively (Fig. 5a). Moreover, the DOS peak positions mapped by the separate ML scheme are much better matched with the DFT peaks than those mapped by the total ML scheme (Fig. 5b,c). Similar improvements are also observed in other bimetallic NP cases (Supplementary Figs. S6–S8).

The main origin of improvement can be explained with the dispersity of DOS patterns, similar to pure NP cases. In the total learning scheme, the average of standard deviation for DOS patterns of the Au–Pt NPs at each energy level is 0.323. This value is much higher than those of Au and Pt atoms in separate learning scheme, which are 0.268 and 0.259, respectively. This trend is also confirmed with the ratio of information for the PCA process (Supplementary Fig. S9). Indeed, the total learning scheme shows much lower reconstruction rates of DOS patterns than those of the separate learning.

### Computational cost for DOS prediction

**Figure 6:**
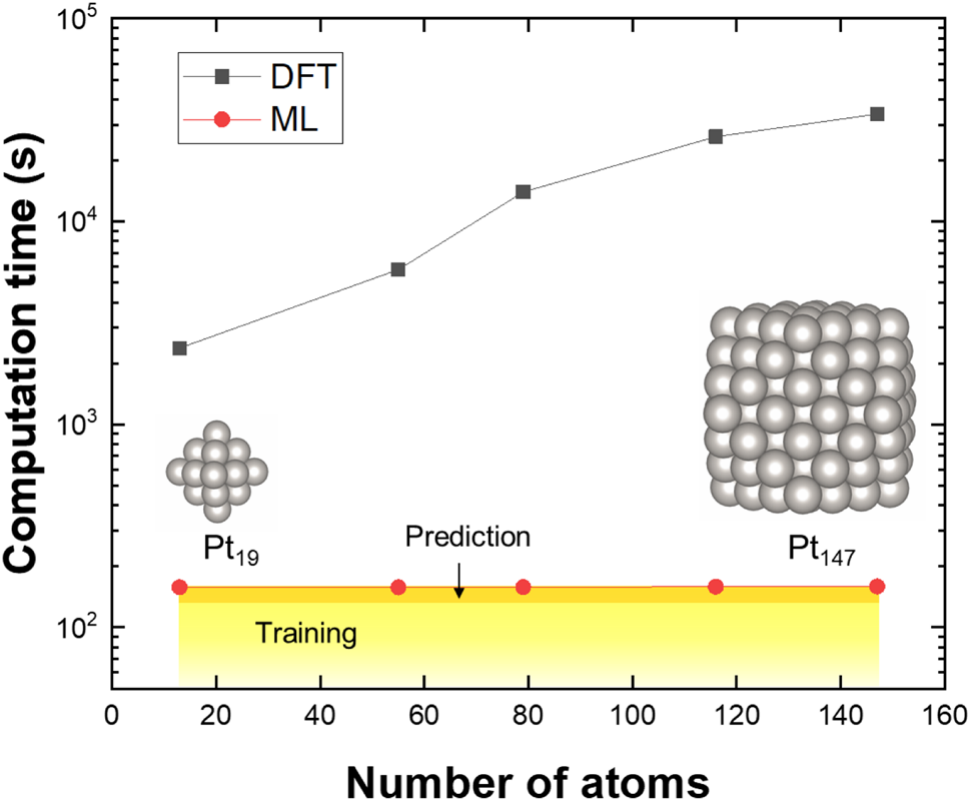
Computational cost of of PCA-CGCNN methods. Comparison of the computation time for calculations of DOS patterns of metallic NPs via DFT (black) and ML (red). For the DFT calculations, a 2.3 GHz 20 core CPU was used. For the PCA-CGCNN methods, a personal computer with a GTX 2070 GPU and i5-9600 K CPU was used, and the computational times were measured as a sum of training and prediction times.

As already mentioned, DFT calculations of NP structures require an extremely increasing computational cost as the NP size increases. Thus, a comparison of computation speeds between DFT and the PCA-CGCNN method is of great interest. With an example of Pt NPs, we benchmark the computational speeds of each method (Fig. 6). Here, the DFT calculations were performed on 20 cores of a 2.3 GHz central processing unit (CPU), while the PCA-CGCNN calculations were performed on a personal computer with a single GTX 2070 graphics processing unit (GPU). In Fig. 6, it is clear that the PCA-CGCNN method is extremely fast compared with the DFT method. For example, for Pt_116_ and Pt_147_ NPs, the DOS calculations via the DFT method take 430 and 570 h, respectively, which are much longer times than those for the PCA-CGCNN method (158 s for Pt_116_ and 159 s for Pt_147_). Here, the computational times for the PCA-CGCNN method are measured as a sum of training and prediction times. The PCA-CGCNN method takes only ~ 160 s (training: ~ 150 s, prediction: < 10 s) for mapping the DOS patterns of NPs irrespective of the sizes of NPs, which is similar to the times reported for the previous PCA-only model^30^. This indicates that the addition of the CGCNN into the PCA method does not sacrifice the computational cost at all; instead, the addition of the CGCNN provides a more flexible and accurate approach. Moreover, as already mentioned, the prediction speed of our PCA-CGCNN scheme is not nearly as affected by the system sizes of NPs when compared with DFT frameworks, indicating that it has a potential for a higher speed than other linear scale methods such as tight binding (TB) or density functional TB (DFTB).

## Conclusion

In conclusion, we have developed the ML model combining PCA and the CGCNN to predict the DOS patterns of various types of NPs with a handful of training sets that can be obtained without great difficulty by the typical DFT frameworks, in which the PCA-CGCNN method is applicable for not only pure NPs but also bimetallic NPs. Comparing different types of NPs, there was a performance change in the ML model, which results from the dispersity difference in DOS patterns of NPs. In particular, for pure NPs case, it originates from the dipole polarizability between Au, Pt, and Pd. Although there is a small loss of accuracy with our PCA-CGCNN method compared to DFT calculations, the prediction speed is much faster than those of typical DFT frameworks. In particular, the prediction speed is not nearly as affected by the system sizes of NPs when compared with DFT frameworks. In this regard, our ML approach can become an option to circumvent DFT calculations, with which one can predict the DOS patterns of actual nanometer-scale NPs mostly synthesized in experiments which remains challenging within DFT frameworks. In this work, our ML model was applied for total DOS with up-spin only. However, with the sufficient DOS pattern data, our model would be readily applicable for the different types of DOS patterns (e.g., down-spin or d-orbital DOS) via a separate learning scheme. Therefore, our approach can be immediately applied to accelerate material design in diverse nanotechnology fields such as catalysis, biomaterials, and optics. Moreover, because our approach provides a flexible framework in handling atomic structures, it can be generally used to explore the correlation between atomic structures and other spectrum-type properties of materials (e.g., X-ray diffraction, X-ray photoelectron spectroscopy, Raman spectroscopy, etc.).

## Supplementary Information

Supplementary Information.

## Data Availability

The implemented PCA-CGCNN framework code and data are available at https://github.com/kihoon-bang/PCA-CGCNN, or from the corresponding authors on request.

## References

[1] Chakraborty I, Pradeep T (2017). Atomically precise clusters of noble metals: Emerging link between atoms and nanoparticles. Chem. Rev..

[2] Kwak K, Lee D (2019). Electrochemistry of atomically precise metal nanoclusters. Acc. Chem. Res..

[3] Wang XX (2018). Ordered Pt3Co intermetallic nanoparticles derived from metal-organic frameworks for oxygen reduction. Nano Lett..

[4] Boles MA, Ling D, Hyeon T, Talapin DV (2016). The surface science of nanocrystals. Nat. Mater..

[5] Pradhan S (2019). High-efficiency colloidal quantum dot infrared light-emitting diodes via engineering at the supra-nanocrystalline level. Nat. Nanotechnol..

[6] Chiba T (2018). Anion-exchange red perovskite quantum dots with ammonium iodine salts for highly efficient light-emitting devices. Nat. Photon..

[7] Li Y (2019). Stoichiometry-Controlled InP-based quantum dots: Synthesis, photoluminescence, and electroluminescence. J. Am. Chem. Soc..

[8] Zhu K (2018). Magnetic nanomaterials: Chemical design, synthesis, and potential applications. Acc. Chem. Res..

[9] Batsaikhan E (2020). Largely enhanced ferromagnetism in Bare CuO nanoparticles by a small size effect. ACS Omega.

[10] Duan X, Chan C, Lin W (2019). Nanoparticle-mediated immunogenic cell death enables and potentiates cancer immunotherapy. Angew. Chem. Int. Ed..

[11] Wang L, Hu C, Shao L (2017). The antimicrobial activity of nanoparticles: Present situation and prospects for the future. Int. J. Nanomed..

[12] Dong Z (2018). Synthesis of hollow biomineralized CaCO_3_–polydopamine nanoparticles for multimodal imaging-guided cancer photodynamic therapy with reduced skin photosensitivity. J. Am. Chem. Soc..

[13] Harmsen S, Wall MA, Huang R, Kircher MF (2017). Cancer imaging using surface-enhanced resonance Raman scattering nanoparticles. Nat. Protoc..

[14] Jung C (2017). Synthesis of chemically ordered Pt3Fe/C intermetallic electrocatalysts for oxygen reduction reaction with enhanced activity and durability via a removable carbon coating. ACS Appl. Mater. Interfaces.

[15] Shin K (2017). Interface engineering for a rational design of poison-free bimetallic CO oxidation catalysts. Nanoscale.

[16] Kim D (2019). Unlocking the potential of nanoparticles composed of immiscible elements for direct H_2_O_2_ synthesis. ACS Catal..

[17] Kim S-Y, Lee HW, Pai SJ, Han SS (2018). Activity, selectivity, and durability of ruthenium nanoparticle catalysts for ammonia synthesis by reactive molecular dynamics simulation: The size effect. ACS Appl. Mater. Interfaces.

[18] Creus J (2018). Ligand-capped Ru nanoparticles as efficient electrocatalyst for the hydrogen evolution reaction. ACS Catal..

[19] Wang C, Yang H, Zhang Y, Wang Q (2019). NiFe alloy nanoparticles with hcp crystal structure stimulate superior oxygen evolution reaction electrocatalytic activity. Angew. Chem. Int. Ed..

[20] Wang H (2019). Disentangling the size-dependent geometric and electronic effects of palladium nanocatalysts beyond selectivity. Sci. Adv..

[21] Kim M (2020). Artificial intelligence to accelerate the discovery of N2 electroreduction catalysts. Chem. Mater..

[22] Verga LG (2018). DFT calculation of oxygen adsorption on platinum nanoparticles: Coverage and size effects. Faraday Discuss.

[23] Balamurugan B, Maruyama T (2005). Evidence of an enhanced interband absorption in Au nanoparticles: Size-dependent electronic structure and optical properties. Appl. Phys. Lett..

[24] Zhang P, Jin W, Liang W (2018). Size-dependent optical properties of aluminum nanoparticles: From classical to quantum description. J. Phys. Chem. C.

[25] Bai L (2016). Explaining the size dependence in platinum-nanoparticle-catalyzed hydrogenation reactions. Angew. Chem. Int. Ed..

[26] Liu Z, Wang G (2017). Shape-dependent surface magnetism of Co-Pt and Fe-Pt nanoparticles from first principles. Phys. Rev. B.

[27] Adhikari K (2018). Benchmarking the performance of plane-wave vs. localized orbital basis set methods in DFT modeling of metal surface: A case study for Fe-(110). J. Comput. Sci..

[28] Brockherde F (2017). Bypassing the Kohn–Sham equations with machine learning. Nat. Commun..

[29] Chandrasekaran A (2019). Solving the electronic structure problem with machine learning. npj Comput. Mater..

[30] Yeo BC, Kim D, Kim C, Han SS (2019). Pattern learning electronic density of states. Sci. Rep..

[31] Takigawa I, Shimizu K-I, Tsuda K, Takakusagi S (2016). Machine-learning prediction of the d-band center for metals and bimetals. RSC Adv..

[32] Umeno Y, Kubo A (2019). Prediction of electronic structure in atomistic model using artificial neural network. Comput. Mater. Sci..

[33] Zuo Y (2020). Performance and cost assessment of machine learning interatomic potentials. J. Phys. Chem. A.

[34] Schleder GR, Padilha ACM, Acosta CM, Costa M, Fazzio A (2019). From DFT to machine learning: Recent approaches to materials science—A review. J. Phys. Mater..

[35] Ramprasad R, Batra R, Pilania G, Mannodi-Kanakkithodi A, Kim C (2017). Machine learning in materials informatics: Recent applications and prospects. npj Comput. Mater..

[36] Zhang Y, Xu X (2021). Predictions of the total crack length in solidification cracking through LSBoost. Metall. Mater. Trans. A.

[37] Zhang Y, Xu X (2021). Machine learning properties of electrolyte additives: A focus on redox potentials. Ind. Eng. Chem. Res..

[38] Zhang Y, Xu X (2020). Solubility predictions through LSBoost for supercritical carbon dioxide in ionic liquids. New J. Chem..

[39] Zhang Y, Xu X (2020). Machine learning modeling of lattice constants for half-Heusler alloys. AIP Adv..

[40] Zhang Y, Xu X (2021). Predictions of adsorption energies of methane-related species on Cu-based alloys through machine learning. Mach. Learn. Appl..

[41] Chu W, Saidi WA, Prezhdo OV (2020). Long-lived hot electron in a metallic particle for plasmonics and catalysis: Ab initio nonadiabatic molecular dynamics with machine learning. ACS Nano.

[42] Zeni C, Rossi K, Glielmo A, Baletto F (2019). On machine learning force fields for metallic nanoparticles. Adv. Phys. X.

[43] Xie T, Grossman JC (2018). Crystal graph convolutional neural networks for an accurate and interpretable prediction of material properties. Phys. Rev. Lett..

[44] Li H (2015). Magic-number gold nanoclusters with diameters from 1 to 3.5 nm: Relative stability and catalytic activity for CO oxidation. Nano Lett..

[45] Mostafa S (2010). Shape-dependent catalytic properties of Pt nanoparticles. J. Am. Chem. Soc..

[46] Hjorth Larsen A (2017). The atomic simulation environment—A python library for working with atoms. J. Phys. Condens. Matter.

[47] Plimpton S (1995). Fast parallel algorithms for short-range molecular dynamics. J. Comput. Phys..

[48] Zhou XW, Johnson RA, Wadley HNG (2004). Misfit-energy-increasing dislocations in vapor-deposited CoFe/NiFe multilayers. Phys. Rev. B.

[49] Kresse G, Furthmüller J (1996). Efficiency of ab-initio total energy calculations for metals and semiconductors using a plane-wave basis set. Comput. Mater. Sci..

[50] Kresse G, Joubert D (1999). From ultrasoft pseudopotentials to the projector augmented-wave method. Phys. Rev. B.

[51] Perdew JP, Burke K, Ernzerhof M (1996). Generalized gradient approximation made simple. Phys. Rev. Lett..

[52] Hammer B, Hansen LB, Nørskov JK (1999). Improved adsorption energetics within density-functional theory using revised Perdew–Burke–Ernzerhof functionals. Phys. Rev. B.

[53] Blöchl PE (1994). Projector augmented-wave method. Phys. Rev. B.

[54] Pack JD, Monkhorst HJ (1977). "Special points for Brillouin-zone integrations"—A reply. Phys. Rev. B.

[55] Srivastava N, Hinton G, Krizhevsky A, Sutskever I, Salakhutdinov R (2014). Dropout: A simple way to prevent neural networks from overfitting. J. Mach. Learn. Res..

[56] Aarons J, Sarwar M, Thompsett D, Skylaris C-K (2016). Perspective: Methods for large-scale density functional calculations on metallic systems. J. Chem. Phys..

[57] Schwerdtfeger P, Nagle JK (2019). 2018 Table of static dipole polarizabilities of the neutral elements in the periodic table. Mol. Phys..

