# Supplementary material for: Accelerated mapping of electronic density of states patterns of metallic nanoparticles via machine-learning
Source: Sci Rep. 2021 Jun 2;11:11604. doi: 10.1038/s41598-021-91068-8 (PMC8173009; doi:10.1038/s41598-021-91068-8)
Supplement: Supplementary file 1 — Supplementary Information. [file 41598_2021_91068_MOESM1_ESM.pdf]

Supplementary Material

Accelerated Mapping of Electronic Density of States Patterns of Metallic Nanoparticles via Machine-Learning

Kihoon Bang,[†] Byung Chul Yeo,[‡] Donghun Kim,[‡] Sang Soo Han,[‡,*] Hyuck Mo Lee[†,*]

[†]Department of Materials Science and Engineering, Korea Advanced Institute of Science and Technology (KAIST), 291 Daehak-ro, Yuseong-gu, Daejeon 34141, Republic of Korea

[‡]Computational Science Research Center, Korea Institute of Science and Technology (KIST), 5 Hwarangno 14-gil, Seongbuk-gu, Seoul 02792, Republic of Korea

*To whom correspondence should be addressed.

E-mail: sangsoo@kist.re.kr (S.S.H.) and hmlee@kaist.ac.kr (H.M.L.)

Construction of nanoparticles (NPs) with asymmetric shapes by molecular dynamics simulations

In the electronic density of states (DOS) database of NPs, we considered not only symmetric but also asymmetric NP structures. To generate the asymmetric NPs, we employed molecular dynamics (MD) simulations in which the LAMMPS program was used. The MD time-step was chosen as 1 fs and the canonical (NVT) ensemble was used. The box size for the MD simulation was 35×35×35 $Å^3$,in which one NP structure was included. The MD procedure is as follows:

1. Prepare a NP cluster composed of M atoms. (M=40, 45, 50)
2. For 200 ps, heat the box up to 900 K which is higher than melting temperature of the cluster. During the heating process, the unsymmetric shapes of NPs can be generated.
3. For 10 ps, rapidly cool down the box to 10 K to maintain the asymmetric shapes.

Then, with the NPs with asymmetric shapes, we calculated their DOS patterns by DFT without ionic relaxation.

Table S1. Properties used in the atom feature vector v_i

**Property**	**Range**	**Unit**	**Category #**
Group number	1, 3 – 12, 15		12
Period number	1 – 6		6
Electronegativity	1.2 – 3.2		10
1st ionization energy	6.5 – 15	eV	10
Electron affinity	-0.8 – 2.4	eV	10
Density	0 – 23	g/cm^3	10
Weight	1 – 240	g/mol	10
Radius	0 – 1.65	Å	10
Atomic volume	6.5 – 18	cm^3/mol	10
Melting point	-260 – 3500	℃	10
Boiling point	-274 – 5600	℃	10
Z_{eff}	1 – 5.3		10
Heat of vaporization	100 – 750	kJ/mol	10
Heat of fusion	6 – 30	kJ/mol	10
Polarizability	25 – 60	Atomic unit	10
Resistivity	0 – 160	10^{-8} Ω·m	10
Atomization energy	250 – 750	kJ/mol	10
Heat capacity	0.12 – 0.57	J/g·K	10
Number of valence electrons	0 – 10		10
Number of d electrons	0 – 10		10

Table S2. Properties used in the bond feature vector $u_{(i,j)}$

**Property**	**Range**	**Unit**	**Category #**
Atom distance	2.4 – 3.4	Å	40

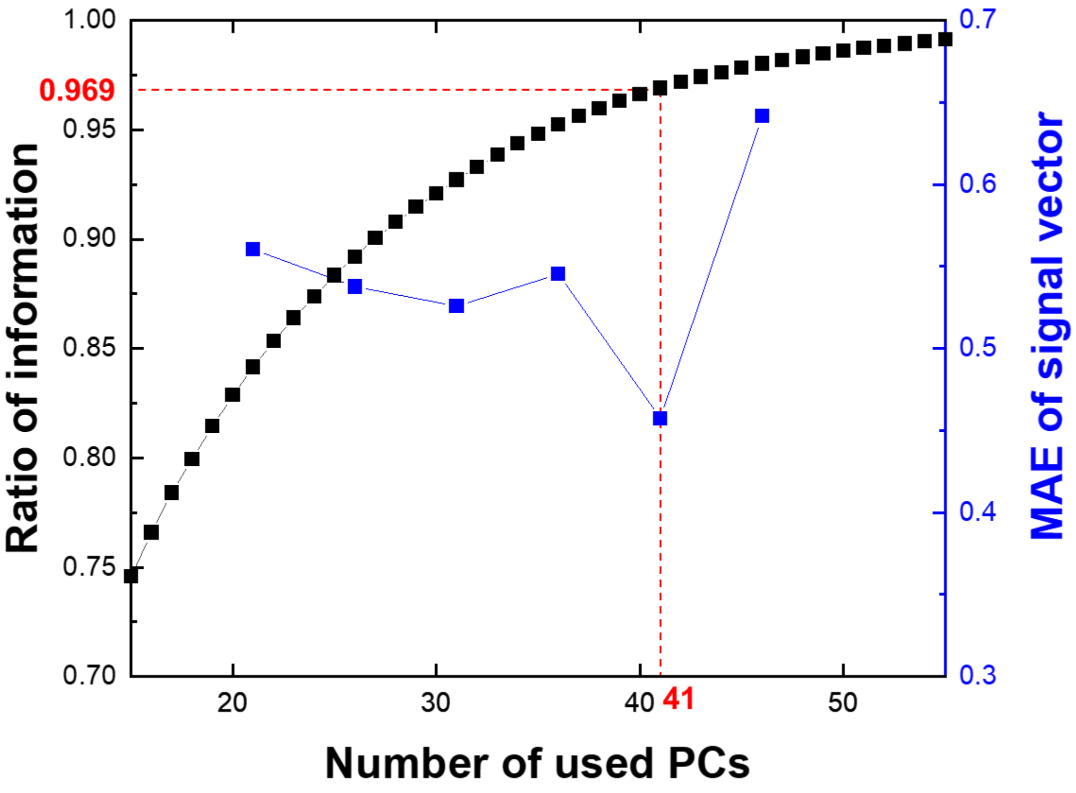

Figure S1. Performance tests of the PCA-CGCNN model for the number of used PCs. Black data indicate the ratio of information of PCA analysis and blue data indicate the mean absolute errors (MAEs) of the signal vectors. The lowest error of the PCA-CGCNN model is observed with 41 PCs.

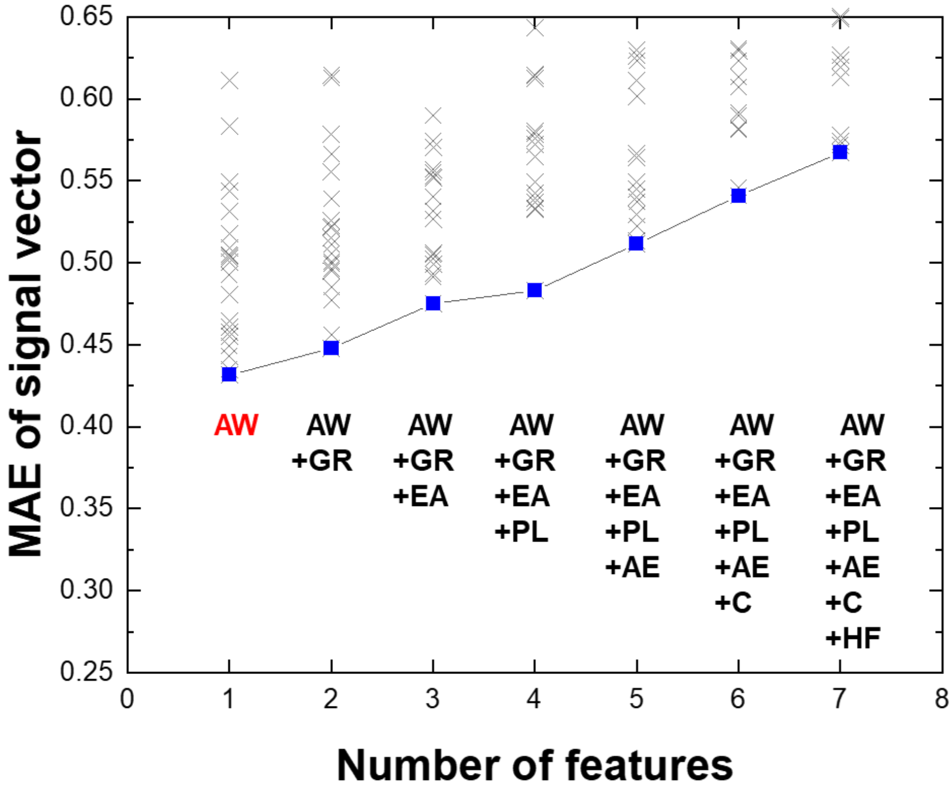

Figure S2. Test for the optimization of material features. MAE values are compared as a function of various feature combinations. Blue points/line denote the minimum value at each feature combination. The best performance is observed with one feature (AW) is used. Here, AW, GR, EA, PL, AE, C, and HF means atomic weight, group number, electron affinity, polarizability, atomization energy, heat capacity, and heat of fusion, respectively.

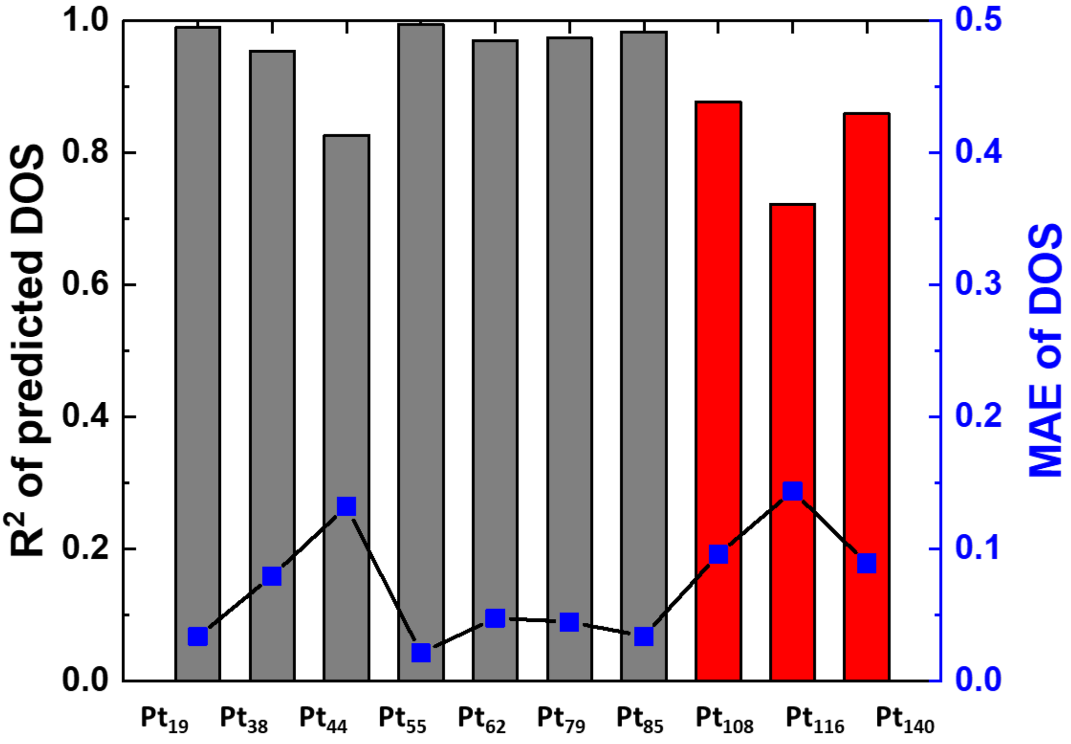

Figure S3. The DOS pattern similarities (R^2 and MAE) between our PCA-CGCNN model and DFT methods. Here, pure Pt NPs are considered. Bars indicate R^2 value and blue squares indicate MAE. Gray bars indicate training data and red bars indicate test data.

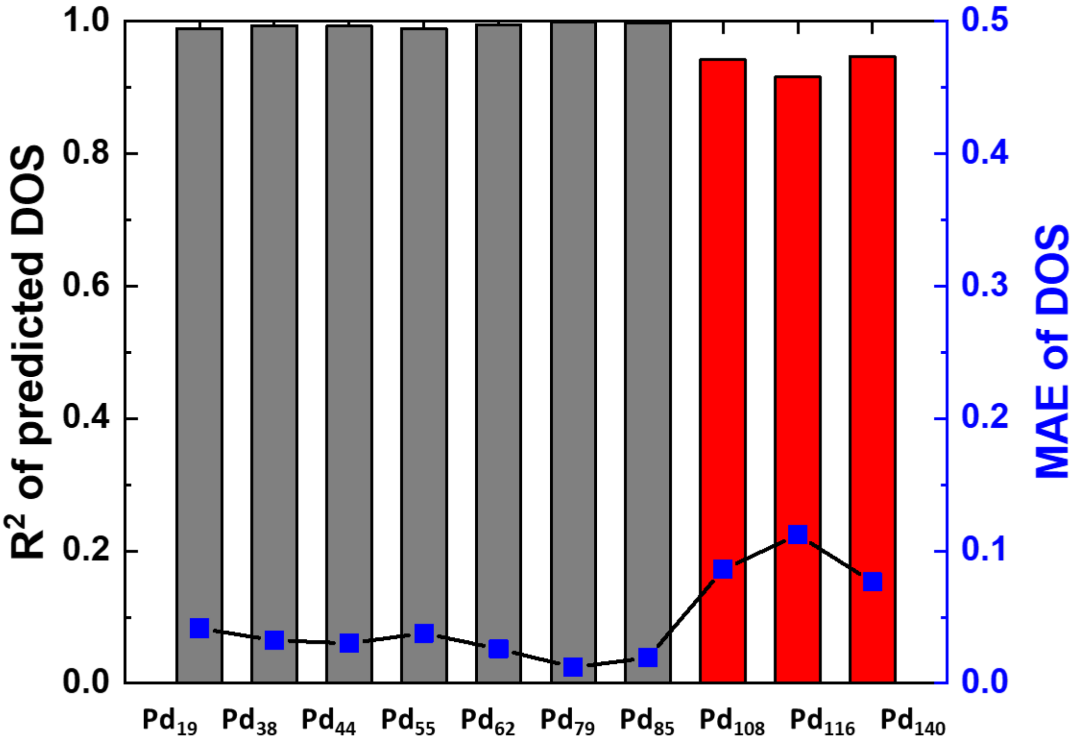

Figure S4. The DOS pattern similarities (R^2 and MAE) between our PCA-CGCNN model and DFT methods. Here, pure Pd NPs are considered. Bars indicate R^2 value and blue squares indicate MAE. Gray bars indicate training data and red bars indicate test data.

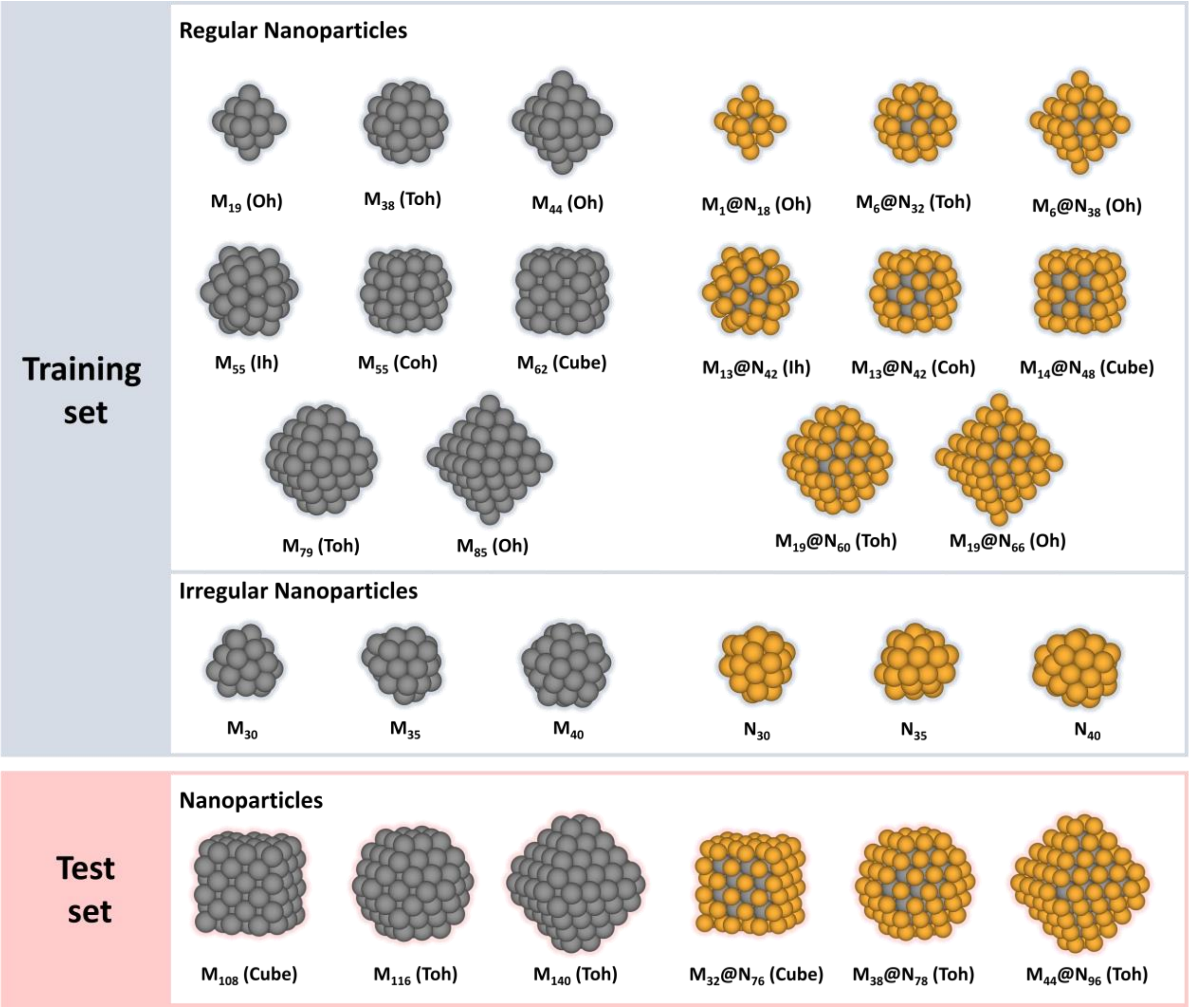

Figure S5. Training and test datasets for DOS prediction of bimetallic NPs. In the NP structures, COh, Ih, Oh, TOh, and Cube indicate cuboctahedral, icosahedral, octahedral, tetraoctahedral, and cubic structures, respectively.

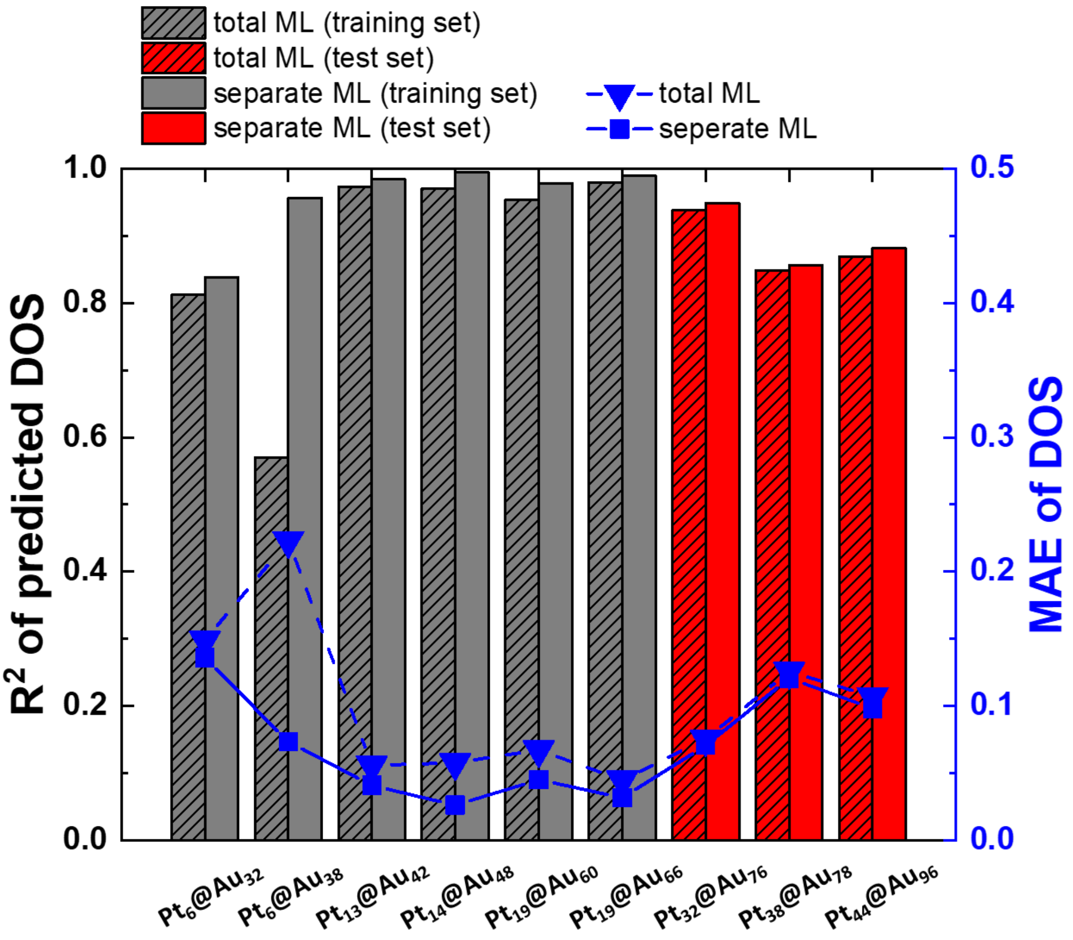

Figure S6. The DOS pattern similarity (R^2 and MAE) between our PCA-CGCNN model and DFT methods. Here, Pt@Au core@shell NPs are considered. Bars indicate R^2 value and blue squares indicate MAE. Gray and red bars indicate training and test data, respectively. In addition, line and solid bars indicate total and separate learning methods, respectively.

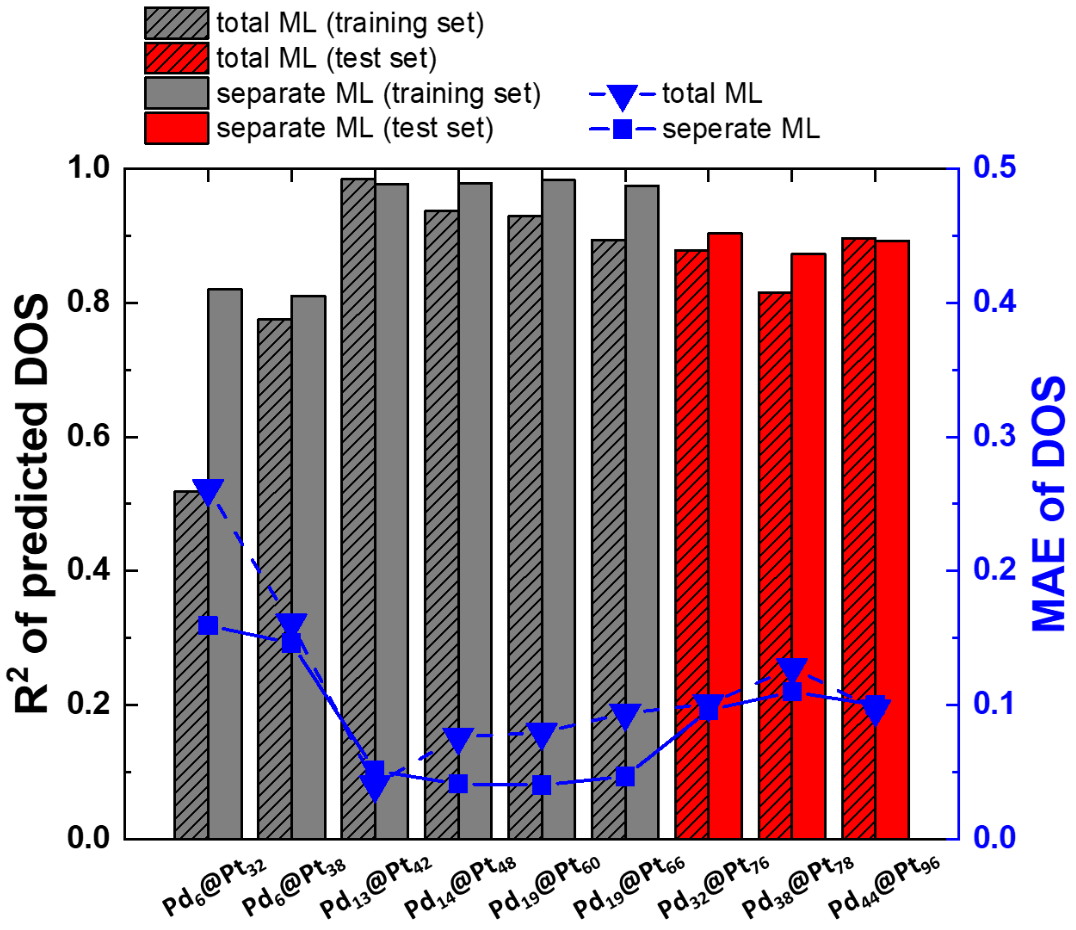

Figure S7. The DOS pattern similarity (R^2 and MAE) between our PCA-CGCNN model and DFT methods. Here, Pd@Pt core@shell NPs are considered. Bars indicate R^2 value and blue squares indicate MAE. Gray and red bars indicate training and test data, respectively. In addition, line and solid bars indicate total and separate learning methods, respectively.

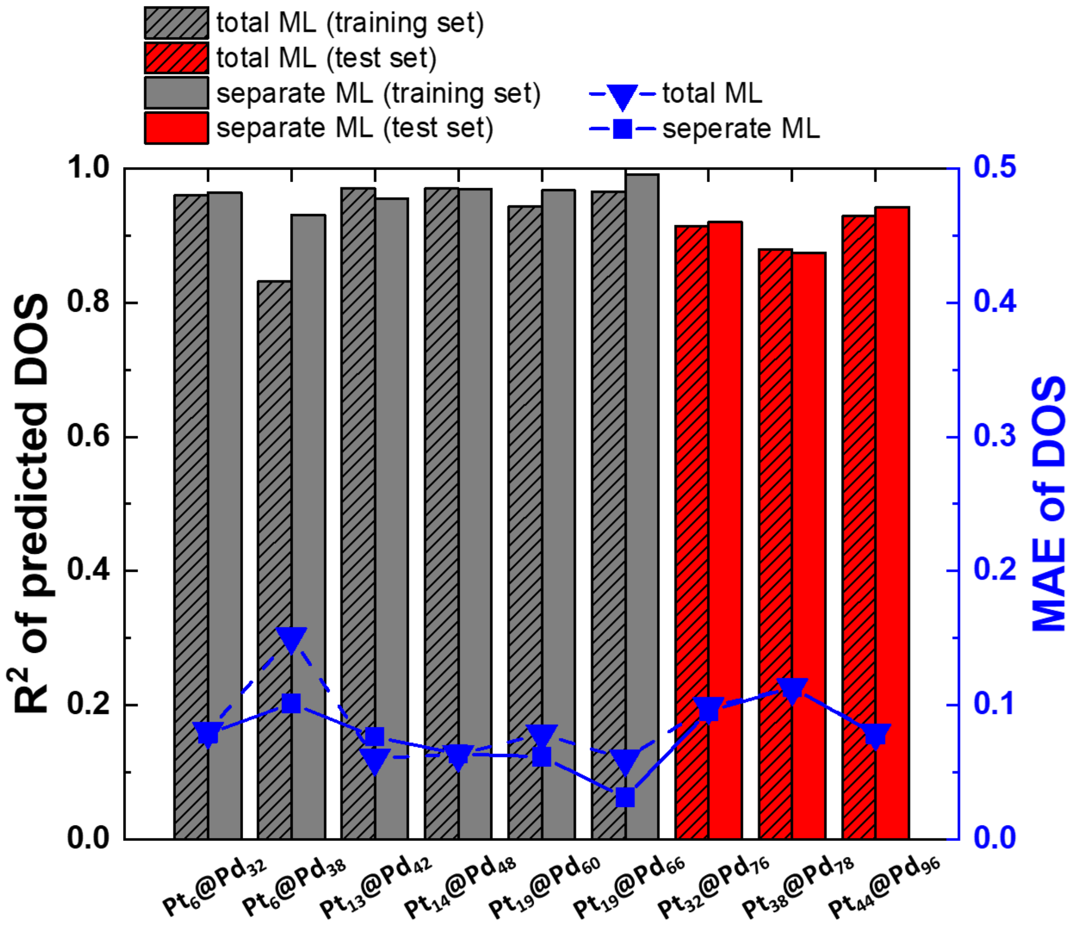

Figure S8. The DOS pattern similarity (R^2 and MAE) between our PCA-CGCNN model and DFT methods. Here, Pt@Pd core@shell NPs are considered. Bars indicate R^2 value and blue squares indicate MAE. Gray and red bars indicate training and test data, respectively. In addition, line and solid bars indicate total and separate learning methods, respectively.

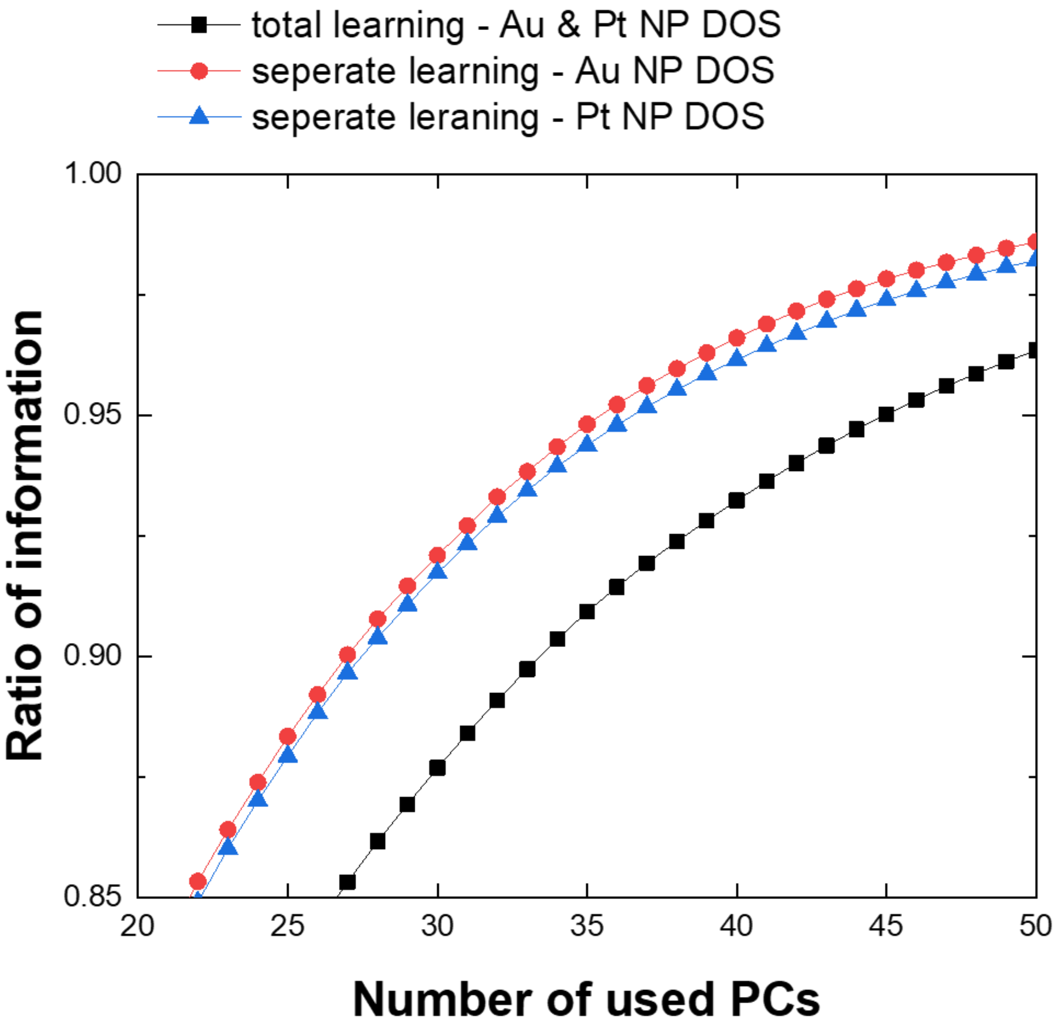

Figure S9. The ratio of information of Au-Pt bimatllic NP DOSs for the total learning and separate learning schemes in PCA. Here, the ratio for the total learning scheme is lower than the separate learning shceme.

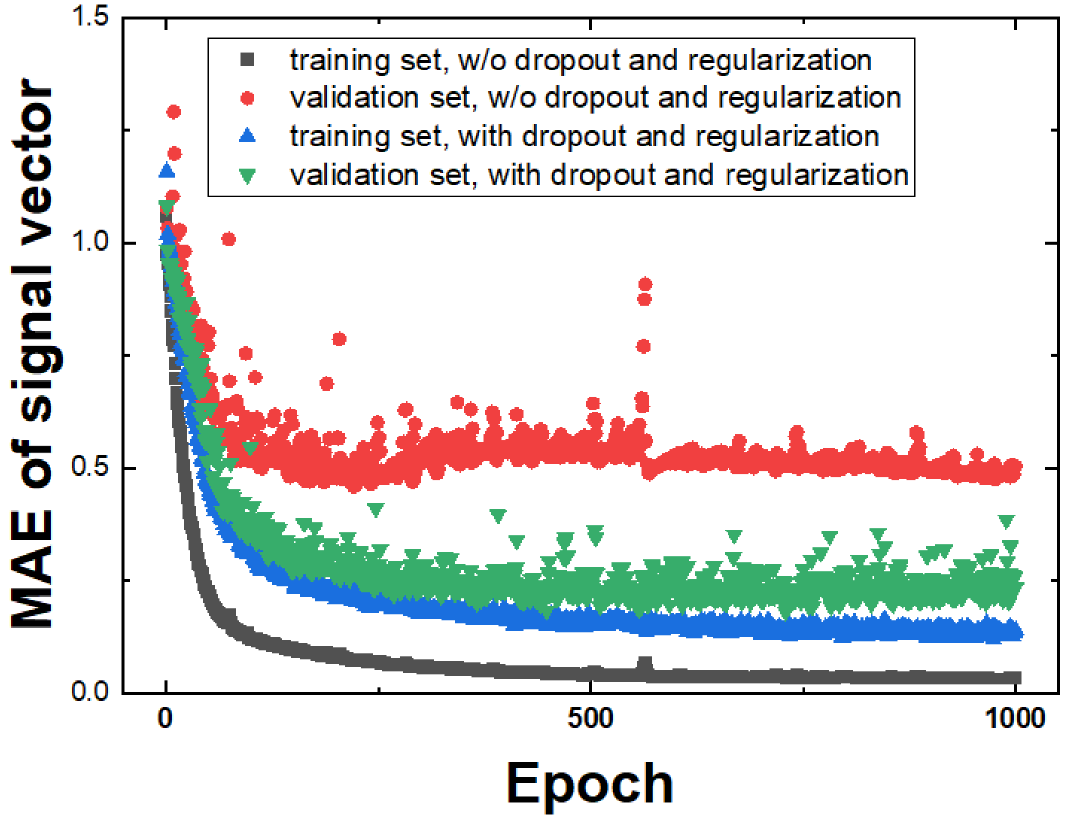

Figure S10. The MAE of signal vector in the training and validation sets during the learning process of PCA-CGCNN. Black and red dots correspond to the results without the dropout and regularization, while blue and green dots correspond to the results with the dropout and regularization.
